# Case report: Beyond arrhythmia: STAR-inspired single-fraction cardiac radioablation for Ewing sarcoma metastasis

**DOI:** 10.3389/fonc.2026.1875162

**Published:** 2026-06-30

**Authors:** Erika Galietta, Silvia Strolin, Lidia Strigari, Francesco Cellini, Savino Cilla, Leonardo Santarelli, Vincenzo Russo, Alessandra Arcelli, Cristian Martignani, Costanza Maria Donati, Alessio G. Morganti, Silvia Cammelli

**Affiliations:** 1 Department of Medical and Surgical Sciences (DIMEC), Alma Mater Studiorum University of Bologna, Bologna, Italy; 2 Radiation Oncology, IRCCS Azienda Ospedaliero-Universitaria di Bologna, Bologna, Italy; 3 Medical Physics, IRCCS Azienda Ospedaliero-Universitaria di Bologna, Bologna, Italy; 4 Fondazione Policlinico Universitario A. Gemelli, IRCCS, UOC di Radioterapia, Dipartimento di Scienze Radiologiche, Radioterapiche ed Ematologiche, Roma, Italy; 5 Istituto di Radiologia, Università Cattolica del Sacro Cuore, Roma, Italy; 6 Medical Physics Unit, Responsible Research Hospital, Gemelli Molise Hospital, Campobasso, Italy; 7 Department of Pediatric and Adult Cardio-Thoracovascular, Oncohematologic and Emergencies Radiology Unit, IRCCS Azienda Ospedaliero-Universitaria di Bologna, Bologna, Italy; 8 Cardiology Unit, Cardio-Thoracic and Vascular Department, IRCCS Azienda Ospedaliero-Universitaria di Bologna, Bologna, Italy

**Keywords:** cardiac metastasis, CARE guidelines, Ewing sarcoma, single-fraction stereotactic body radiotherapy, STAR regimen

## Abstract

**Introduction:**

Stereotactic arrhythmia radioablation (STAR) has emerged as an effective non-invasive treatment for refractory ventricular arrhythmias, but its ablative single-fraction regimen may also be relevant beyond electrophysiologic indications. We report the application of a STAR-inspired 25 Gy single-fraction cardiac radioablation approach for right-ventricular metastasis from Ewing sarcoma, presented as a CARE-compliant case report.

**Case presentation:**

A 26-year-old man with a history of limb Ewing sarcoma developed an incidentally detected right-ventricular mass during routine staging. After partial surgical debulking, multimodal imaging confirmed a 17 × 15 mm residual intracavitary lesion. Because complete surgical resection was not technically feasible, frameless stereotactic body radiotherapy (SBRT) was delivered in a single 25 Gy fraction using STAR workflow principles. Treatment was completed without complication. At 12-month MRI the lesion was reduced (8 × 10 mm), and subsequent imaging confirmed persistent local cardiac control. At 31 months after STAR, the patient remained free of cardiac symptoms and signs of cardiac disease progression.

**Conclusion:**

Adapting the STAR single-fraction regimen for oncologic cardiac targets is feasible and provided durable control with no observed cardiac toxicity. This experience supports further evaluation of STAR-inspired SBRT for unresectable cardiac tumors.

## 1 Introduction

Primary and secondary cardiac tumors are rare: autopsy studies estimate an incidence of 0.002-0.3% for primary lesions and up to 18% for metastatic involvement (1, 2). Surgery remains the treatment of choice when technically feasible, but many patients are unsuitable because of tumor location, comorbidities, or systemic disease burden.

Stereotactic arrhythmia radioablation (STAR) has recently expanded the therapeutic landscape for patients with refractory ventricular tachycardia by demonstrating that a single 25 Gy fraction can be delivered safely and effectively to a highly mobile intracardiac target (3–5). Although this strategy was developed for arrhythmic substrates rather than cancer, the technical and radiobiological principles underlying STAR are potentially attractive for selected oncologic scenarios, particularly when a cardiac lesion cannot be completely resected and conventional local options are limited.

In this context, applying a STAR-inspired ablative regimen to a malignant cardiac target may represent a meaningful extension of the paradigm beyond its standard indication. We therefore present a CAse REport Guidelines (CARE)-compliant (6) case report of right-ventricular (RV) metastasis from Ewing sarcoma treated with single-fraction cardiac radioablation using a STAR-derived approach. A compiled CARE checklist is available in **Supplementary Table 1**.

## 2 Patient information

A previously healthy 26-year-old man first noticed a painful swelling in his left thigh in October 2021. Magnetic Resonance Imaging (MRI) performed in March 2022 revealed a 12.5 × 6 cm mass within the biceps femoris; core biopsy confirmed Ewing sarcoma with EWSR1 (Ewing sarcoma breakpoint region 1) rearrangement, the canonical molecular hallmark of these tumors, and diffuse CD99 (Cluster of differentiation 99) positivity, a typical immunohistochemical feature supporting the diagnosis. Baseline Fluorine-18 Fluorodeoxyglucose Positron Emission Tomography (^18F^-FDG PET) identified inguinal/iliac nodal and multiple pulmonary metastases.

A routine pre-chemotherapy Transthoracic Echocardiogram (TTE) in April 2022 unexpectedly showed a mobile RV mass. The lesion was surgically debulked a few days later, but a macroscopic residual lesion remained because complete resection was not technically feasible. Over the next year the patient underwent multi-agent chemotherapy, external-beam radiotherapy to the thigh, and finally high-dose Busulfan-Melphalan with autologous stem-cell rescue. Serial cardiologic assessments, including electrocardiography performed before each new chemotherapy course, consistently showed no functional abnormalities or conduction disturbances. Follow-up cardiac MRI in January 2023 confirmed residual RV disease.

## 3 Clinical findings

In June 2023, the patient was evaluated in the radiotherapy department for possible irradiation of the cardiac metastasis. Baseline vital signs were stable (blood pressure 118/72 mmHg, heart rate 64 bpm). There were no murmurs, gallops or signs of heart failure. Laboratory tests showed normal cardiac biomarkers. In addition, serial electrocardiograms (ECG) performed throughout the oncologic course, including before each subsequent chemotherapy line, remained unremarkable and showed no evidence of treatment-related functional cardiac impairment or clinically relevant conduction abnormalities. The Eastern Cooperative Oncology Group (ECOG) performance status was 1, meaning the patient was fully ambulatory and capable of light daily activities, with only mild limitations in strenuous physical effort.

## 4 Timeline

**Table:** 

Date	Event
Oct 2021	Onset of painful swelling in left thigh
Mar 2022	MRI 12.5 × 6 cm thigh lesion; biopsy → Ewing sarcoma (EWSR1+, CD99+)
Apr 2022	Pre-chemotherapy echocardiogram detects RV mass; subtotal surgical debulking performed days later, with macroscopic residual disease left in place because complete resection was not technically feasible
May 2022	Progression of pulmonary, nodal and local disease → systemic chemotherapy; pre-treatment ECG showed no functional abnormalities
Sep 2022	External RT to thigh: 42 Gy/28 BID (twice-daily) fractions + 12 Gy/8 fractions boost
Dec 2022	High-dose Busulfan + Melphalan with autologous stem-cell rescue; pre-treatment ECG remained unremarkable
Mar 2023	Cardiac MRI shows residual RV lesion (17 × 15 mm)
Jun 2023	Single-fraction STAR-inspired SBRT 25 Gy to RV target; inpatient monitoring uneventful
Jul 2023-Aug 2024	Multiple chemo/RT courses for lung lesions; no thoracic surgery indicated
Sep 2023	2.5-month (after STAR) MRI: RV lesion stable
Jun 2024	12-month (after STAR) MRI: tumor shrinkage (8 × 10 mm)
Jul 2024	CT shows new lung progression → additional chemotherapy; pre-treatment ECG showed no new abnormalities
Nov 2024	17-month MRI: RV lesion stable; no detectable cytotoxic damage
Dec 2024	New RT course on lung lesions
Jan 2025	18-month PET-FDG: no evidence of myocardial lesions; no cardiac recurrence detected on transesophageal echocardiogram
Feb 2025	SBRT on left occipitoparietal brain lesion
Mar 2025	Progression of pulmonary disease → systemic chemotherapy; pre-treatment ECG remained negative for functional or conduction abnormalities
Nov 2025	Re-irradiation of the left occipitoparietal brain lesion
Jan 2026	Latest follow-up MRI (31 months after STAR): persistent RV control; patient asymptomatic and fully active; Reirradiation of the left occipitoparietal brain lesion; Progression of pulmonary disease→ systemic chemotherapy switched to ifosfamide (ongoing)

## 5 Diagnostic assessment

Cardiac MRI characterized the intracavitary lesion as 17 × 15 mm with intermediate T1 and hyperintense T2 signal and avid FDG uptake (SUVmax 6.4) on FDG-positron emission tomography. No additional cardiac lesions were identified.

## 6 Therapeutic intervention

After multidisciplinary discussion the patient was scheduled for single-fraction SBRT adapted from the STAR workflow. Cine-MRI was performed to characterize the lesion and assess cardiac motion. The residual lesion was contoured on MRI and registered to the planning CT using elastic image registration to minimize registration uncertainties between the two modalities. Respiratory-gated CT simulation and 4-D sorting quantified target motion at < 3 mm, allowing a 5 mm isotropic margin to generate the planning target volume (PTV). No ITV was generated; the PTV was obtained by directly expanding the GTV/CTV (GTV = CTV = 4.9 cc) with a 5 mm isotropic margin, considered adequate given the limited motion quantified on 4D-CT and the accuracy provided by the elastic image registration workflow. Treatment was delivered on an Elekta Versa-HD linac (Elekta, Stockholm, Sweden) using two 6 FFF (Flattening Filter Free) full arcs (MU per fraction: 2015.6 and 2263.3), in breath hold. A cone-beam CT (CBCT) was performed immediately before treatment delivery to verify target localization and ensure accurate radiation delivery. The prescription of 25 Gy to the 80% isodose achieved 98.8% PTV coverage (PTV: 47 ml) while respecting organ-at-risk constraints derived from the STAR literature and adapted from the CORSAIR summary by Bisello et al. (7). PTV dosimetric parameters were as follows: Dmax = 25.4 Gy; D0.5cc = 24.97 Gy; Dmin=22.97 Gy; Dmean = 24.52 Gy. The conformity index (CI _ICRU_=V_RI_/PTV) was 1.06, indicating excellent plan conformity; the gradient index (GI_Paddick_=V50%/PTV) was 3.54. The homogeneity index [HI_ICRU_=(D_2%_-D_98%_)/D_50%]_ was 0.043, confirming a highly homogeneous dose distribution within the PTV. Treatment plan with dose distribution is shown in **Figure 1**. The complete set of dose-volume constraints adopted for treatment planning, together with the corresponding patient-specific dosimetric values, is reported in **Supplementary Table 2**. Real-time six-lead ECG monitoring and pulse oximetry showed no acute arrhythmias or hemodynamic instability.

**Figure 1:**
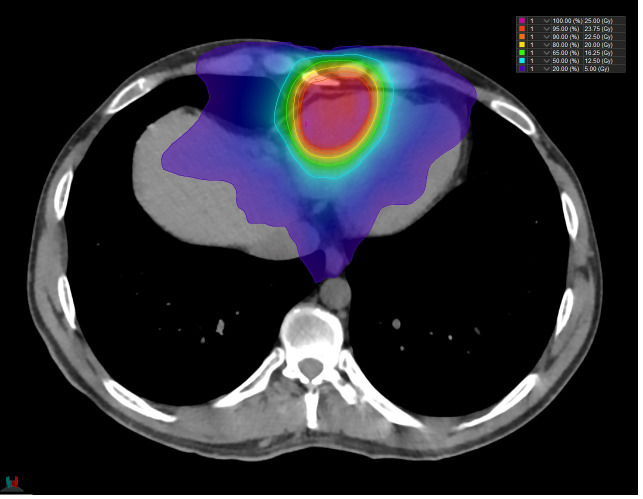
Treatment plan with dose distribution.

## 7 Follow-up and outcomes

The patient was observed overnight and discharged the next morning without complications. No early toxicities were reported at 2-week and 2-month visits. Cardiac MRI at 12 months demonstrated reduced lesion size (8 × 10 mm) and no new late gadolinium enhancement suggestive of radiation-induced cardiomyopathy. Quantitative functional assessment at this time point revealed a left ventricular ejection fraction (LVEF) of 54% and a right ventricular ejection fraction (RVEF) of 50%, both within normal limits according to established reference ranges. The MRI at 17 months confirmed stability of the cardiac lesion without evidence of treatment-induced cytotoxic damage. At 31 months after STAR, there was still no clinical, ECG, or MRI evidence of cardiac functional impairment, and no signs of progression at the treated cardiac site. **Figure 2** shows MRI of the treated lesion before treatment (a), 12 months after treatment (b) and 31 months after treatment (c). The patient remains asymptomatic and continues systemic therapy for extracardiac disease. Throughout follow-up, serial electrocardiographic evaluations remained stable and showed no evidence of functional deterioration or new conduction abnormalities.

**Figure 2:**
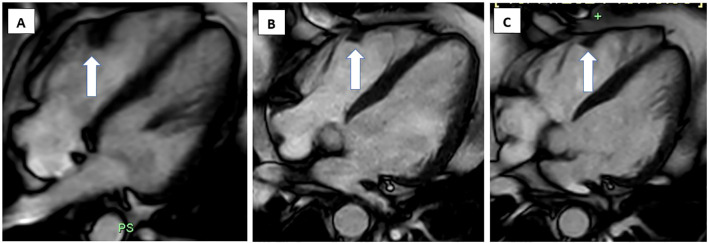
Cardiac magnetic resonance imaging (CMR) in the 4-chamber long-axis view showing the right ventricular lesion at three time points: 2 months before treatment initiation [**(A)** 17 x 10 mm], 12 months after treatment [**(B)** 10 x 8 mm], and 31 months after treatment [**(C)** 10 x 7 mm].

## 8 Discussion

This case illustrates how principles gained from STAR can be leveraged for rare oncologic indications. The decision to prescribe 25 Gy in a single fraction was informed by the extensive STAR experience, where similar doses achieve > 80% VT burden reduction with low toxicity (3–5). Our patient tolerated the regimen well and exhibits durable local control beyond 31 months, with no evidence of cardiac functional impairment or cardiac progression, a meaningful timeframe given the historically poor prognosis of metastatic Ewing sarcoma.

**Table 1** summarizes the limited but growing body of literature on cardiac SBRT (8–14). Most previous reports treated heterogeneous histologies with multi-fraction regimens of 20–36 Gy in three to five fractions. Only two publications, Jumeau et al. (12) and Shah et al. (13), adopted single-fraction schedules, and Shah et al. already reported the use of 25 Gy in a single fraction for recurrent cardiac sarcoma in the re-irradiation setting. However, an important difference should be emphasized: in the report by Shah et al., the target was atrial, whereas in our case the treated lesion was located in the RV. This distinction is clinically relevant because atrial targeting is the conventional anatomical substrate of STAR in arrhythmia treatment, while its application to a ventricular metastatic sarcoma represents, to our knowledge, a unique oncologic use of the STAR paradigm. Our case is therefore not the first sarcoma treated with 25 Gy in a single fraction, but it appears to be the first report of STAR-inspired single-fraction radioablation delivered to a ventricular metastatic sarcoma target. Compared with other available series (15–20), we achieved durable radiological control beyond 31 months without any acute or late cardiac toxicity and without evidence of cardiac functional impairment, despite delivering a markedly ablative single dose to an intracavitary ventricular lesion. Indeed, assuming an α/β ratio of 4 for sarcomas, delivering 25 Gy in a single fraction corresponds to an equivalent dose in 2-Gy fractions (EQD2) of 120.8 Gy, unequivocally an ablative dose. Our experience therefore supports further exploration of STAR-inspired single-fraction SBRT for carefully selected cardiac tumors, particularly when the target is ventricular and complete surgical resection is not feasible.

**Table 1:** Summary of published SBRT experiences for cardiac/paracardiac tumors.

First author/year	Aim/background	Materials & methods	Key results	Authors’ conclusions
Bonomo P, 2013 (8)	Evaluate acute toxicity and local control of SBRT for cardiac/paracardiac metastases	Retrospective series, 16 patients (2009-2011). Dose 36 Gy/3 fractions in 15 patients, 30 Gy/3 fractions in 1 intramyocardial lesion	1 CR, 6 PR, 5 SD; 4 local failures (median 5.2 months). No cardiologic symptoms or ECG changes	SBRT is safe and offers effective local control for cardiac/paracardiac metastases
Bonomo P, 2015 (9)	Test feasibility of fiducial-guided CyberKnife SBRT for primary/secondary cardiac tumors	3 patients (2 recurrent angiosarcoma, 1 melanoma metastasis). 24 Gy/3 fractions or 30 Gy/5 fractions, fiducial tracking	Partial response in both angiosarcomas; stable disease in metastasis. No local progression or toxicity at 6 months MRI	CyberKnife SBRT is feasible and prevents early local progression in selected patients
Dhar A, 2021 (10)	Palliative SBRT for symptomatic myocardial RCC metastasis causing arrhythmia	Single patient; 30 Gy/5 fractions planned with 4D CT + gated MRI fusion	Lesion radiologically stable at 2 months; symptom relief; patient later died of distant progression	SBRT can control symptoms and stabilize isolated cardiac metastases
Gabani P, 2019 (11)	Manage hemodynamic instability from right-atrial primary angiosarcoma with SBRT + paclitaxel	17-year-old female; 30 Gy/5 fractions concurrent paclitaxel	Excellent initial tumor and systemic response; pulmonary progression later; alive at 6 months	SBRT achieves excellent local control, but systemic failure remains major issue
Jumeau R, 2019 (12)	Curative-intent SBRT for oligometastatic NSCLC cardiac metastasis revealed by electrical storm	Single patient; CyberKnife 20 Gy/1 fraction; tracked on pacemaker lead; followed by thoracic chemoradiotherapy	Complete metabolic and radiological response at 3 and 6 months; no VT recurrence, AV block, or toxicity at 9 months	Cardiac SBRT is a promising curative option in selected oligometastatic patients
Shah SN, 2023 (13)	Salvage radiosurgery after failure of prior 72 Gy/15 fractions SBRT in recurrent cardiac sarcoma	53-year-old female; single-fraction 25 Gy CyberKnife re-irradiation to an atrial target	Partial response, no acute toxicity; eventual distant failure, no in-field progression; deceased 16 months after SBRT	Single-fraction salvage radiosurgery can control locally progressive cardiac sarcoma
Michalet M, 2024 (14)	Treat intracardiac metastasis from adrenocortical carcinoma with MR-Linac	65-year-old female; ViewRay MRIdian, 30 Gy/5 fractions with daily adaptive planning	Partial response evident by 3 months, maintained at 6 months; no cardiac dysfunction or other toxicity	MR-guided SBRT is safe and allows precise treatment of highly mobile cardiac lesions
Current report, 2025	Apply single-fraction STAR-inspired SBRT to a metastatic right-ventricular lesion after multimodality therapy	26-year-old male; Elekta Versa-HD, 25 Gy/1 fraction to a ventricular target; PTV 47 ml, coverage 98.8%	No cardiac progression and no cardiac functional impairment at 31 months; no acute or late toxicity; systemic disease managed with further chemotherapy ± SBRT	STAR-inspired single-fraction SBRT to a ventricular metastatic sarcoma target is feasible and yielded durable local control.

ACC, adrenocortical carcinoma; AV, atrioventricular; CR, complete response; CT, computed tomography; EDGE, Varian EDGE radiosurgery system; fx, fractions; mo, months; MRI, magnetic resonance imaging; MR-Linac, MRI-guided linear accelerator; NSCLC, non-small-cell lung cancer; pts, patients; PR, partial response; PTV, planning target volume; RCC, renal cell carcinoma; SBRT, stereotactic body radiotherapy; SD, stable disease; SRS, stereotactic radiosurgery; STAR, stereotactic arrhythmia radioablation; VT, ventricular tachycardia.

Single-fraction delivery confers logistical advantages and limits inter-fraction motion uncertainty compared with multi-fraction cardiac SBRT series that typically use 30 Gy in three to five fractions;however, the potential interplay effect between VMAT dynamic delivery and cardiorespiratory motion should be acknowledged as an inherent limitation of this approach. Although radiobiological equivalence cannot be assumed, pre-clinical data suggest that high single doses may trigger vascular damage (21) and immunogenic cell death (22), both potentially advantageous in a relatively radio-resistant sarcoma substrate; whether these mechanisms contributed to the favorable outcome observed in our patient remains speculative.

The main limitations are the single-patient nature of this report and the short follow-up regarding very late cardiac effects. Nevertheless, the favorable toxicity profile observed in STAR cohorts provides reassuring context (3–5). In addition, reporting the full organ-at-risk dosimetry against established STAR-derived constraints may improve the interpretability and reproducibility of this experience; for this reason, the planning constraints used in our case are detailed in **Supplementary Table 2**. Prospective registries specifically capturing oncologic cardiac SBRT cases are urgently needed to define optimal dose-volume constraints and long-term outcomes.

## 9 Patient perspective

The patient expressed relief at avoiding an additional thoracotomy and was pleased to resume regular activities the day after treatment. He described a sense of reassurance knowing that the cardiac lesion is controlled without ongoing medication.

## Data Availability

The original contributions presented in the study are included in the article/**Supplementary Material**. Further inquiries can be directed to the corresponding author.
