# Supplementary material for: Case report: Beyond arrhythmia: STAR-inspired single-fraction cardiac radioablation for Ewing sarcoma metastasis
Source: Front Oncol. 2026 Jun 30;16:1875162. doi: 10.3389/fonc.2026.1875162 (PMC13364579; doi:10.3389/fonc.2026.1875162)
Supplement: Supplementary file 1 [file SupplementaryFile1.docx]

**SUPPLEMENTARY MATERIAL**

**Supplementary table S1:** CAse REport Guidelines checklist

| **CARE Item** | **Requirement** | **Addressed in Manuscript (Section)** |
| --- | --- | --- |
| Title | Identify as case report and main phenomenon | Title page |
| Key words | Include “case report” plus 2-5 keywords | Title page, Keywords line |
| Abstract | Structured summary (intro, patient info, interventions, outcomes, conclusion) | Abstract |
| Introduction | Brief context and rationale | 1. Introduction |
| Patient information | Demographics, main concerns, history, prior interventions | 2. Patient Information |
| Clinical findings | Relevant exam findings | 3. Clinical Findings |
| Timeline | Chronology of clinical course | 4. Timeline |
| Diagnostic assessment | Methods, challenges, diagnosis, prognosis | 5. Diagnostic Assessment |
| Therapeutic intervention | Type, administration, modifications | 6. Therapeutic Intervention |
| Follow-up and outcomes | Outcomes, follow-up tests, adherence, adverse events | 7. Follow-up and Outcomes |
| Discussion | Strengths, limitations, relevant literature, rationale, lessons | 8. Discussion |
| Patient perspective | Patient’s point of view | 9. Patient Perspective |
| Informed consent | Statement of written consent | 10. Informed Consent |

**Supplementary table S2.** Treatment planning parameters and STAR-derived dose-volume constraints (modified from Bisello et al. 2022 [7].

| Organ / structure | Constraint for single-dose hypofractionation | Patient value |
| --- | --- | --- |
| LAD | Dmax(0.03 cm^3^) ≤ 12 Gy | Dmax(0.03 cm^3^) = 9 Gy |
| Aorta | Dmax(0.1 cm^3^) ≤ 15.4 Gy | Dmax (0.1 cm^3^) = 5.1 Gy |
| Esophagus | Dmax(0.1 cm^3^) ≤ 15.4 Gy | Dmax(0.1 cm^3^) = 6.2 Gy |
| Heart-PTV | Dmax(0.1 cm^3^) ≤ 22 Gy | Dmax(0.1 cm^3^) = 22 Gy |
| CxCA | Dmax(0.03 cm^3^) ≤ 12 Gy | Dmax(0.03 cm^3^) = 10.5 Gy |
| LMCA | Dmax(0.03 cm^3^) ≤ 12 Gy | Dmax(0.03 cm^3^) = 8 Gy |
| Liver | V9.1Gy < 700 cm^3^ | V9.1Gy = 18 cm^3^ |
| Lung | Dmean < 8 Gy | Dmean = 0.8 Gy |
| Proximal bronchus | Dmax(0.1 cm^3^) ≤ 20 Gy | Dmax(0.1 cm^3^) = 0.25 Gy |
| RCA | Dmax(0.03 cm^3^) ≤ 12 Gy | Dmax(0.03 cm^3^) = 11.5 Gy |
| Skin | Dmax(0.01 cm^3^) < 26 Gy | Dmax(0.01 cm^3^) = 16 Gy |
| Spinal cord | Dmax(0.035 cm^3^) < 14 Gy | Dmax(0.035 cm^3^) = 3.7 Gy |
| Superior vena cava | Dmax(0.1 cm^3^) ≤ 15.4 Gy | Dmax(0.1 cm^3^) = 0.2 Gy |
| PTV V90% (22.5 Gy) | - | 100% |
| PTV V95% (23.75 Gy) | - | 98.82% |
| PTV V98% (24.5 Gy) | - | 66.15% |

**Abbreviations:** CxCA, left circumflex coronary artery; DMAX, maximum dose; Gy, Gray; LAD, left anterior descending coronary artery; LMCA, left main coronary artery; PTV, planning target volume; RCA, right coronary artery. All dose values reported in this table are expressed in physical dose and have not been converted to equivalent dose in 2Gy fractions (EQD2).
